# Maternal Humming during Kangaroo Care: Effects on Preterm Dyads’ Physiological Parameters

**DOI:** 10.3390/children11030373

**Published:** 2024-03-21

**Authors:** Maria Eduarda Salgado Carvalho, João Manuel Rosado Miranda Justo

**Affiliations:** 1 Center for Sociology and Musical Aesthetics Studies (CESEM—NOVA FCSH), 1069-061 Lisbon, Portugal; 2 Faculty of Psychology, University of Lisbon, 1649-004 Lisbon, Portugal; jjusto@psicologia.ulisboa.pt

**Keywords:** preterm dyads, physiological parameters, kangaroo care, maternal humming, melodic contours

## Abstract

Humming is probably more effective than speech for improving mothers’ cardiorespiratory function and infants’ self-regulation. We intend to understand the effects of (1) maternal humming vs. speech on preterm infants’ physiological parameters, (2) maternal humming vs. speech on mothers’ physiological parameters, and (3) humming melodic contours and the process of the lengthening of the final note on preterm infants’ physiological parameters. This study was designed as a single-group repeated measures study, using microanalytical methodology (ELAN software version 4.9.4), with a protocol (silent baseline/speech, humming/silence/humming, or speech/silence) applied to preterm dyads (N = 36). Audio and video observations were recorded. Infants’ and mothers’ heart rates (HR) and O_2_ saturations were observed once a minute. The proportion of O_2_ saturation relative to HR (Prop. O_2_ saturation/HR) was estimated for both partners during the protocol. We found that the infants’ HR mean was significantly lower during humming (*p* = 0.028), while a significantly higher Prop. O_2_ saturation/HR ratio was recorded during humming for infants (*p* = 0.027) and mothers (*p* = 0.029). The duration of sinusoidal contours, together with the lengthening of the final note, predicts infants’ Prop. O_2_ saturation/HR ratio. Musical features of humming seem to improve the physiological stability of preterm infants during kangaroo care.

## 1. Background

During the transition between prenatal and neonatal life, the autonomic nervous system (ANS) plays a crucial role in cardiovascular and respiratory functioning [1]. A primary goal of neonatal care for preterm infants is to ensure the stability of ANS [1]. This is achieved through neuroprotective individualized neonatal care centered on infants’ neurodevelopmental needs. NIDCAP (the Newborn Individualized Developmental Care and Assessment Program) focuses on a detailed reading of each infant’s behavioral cues, helping their self-regulation. According to the NIDCAP, environment and care are adapted to enhance each infant’s strengths and self-regulation collaboratively with parents, who play the primary role in ensuring infants’ development [2].

In the NIDCAP approach, kangaroo mother care is one of the multimodal sensory experiences provided in the Neonatal Intensive Care Unit (NICU) through the early skin-to-skin contact between mothers and preterm infants [2]. Kangaroo mother care has been shown to have benefits for preterm infants’ self-regulation, as well as for maintaining the intimate connection between mother and infant [3]. This is achieved through an ANS balance that impacts infants’ physiological stability [2].

Compared to the original vertical positioning method, the “kangaroo supported diagonal flexion positioning” (KSDFP) protocol [4] replaces the previous vertical position of the preterm infant via diagonal positioning, improving mother–infant gaze contact. Furthermore, it ensures preterm infants’ physiological stability, improving the contingent interaction between mother and infant [5], and, thus, it has been underlined as a promissory method for improving the understanding of the innate basis of intersubjectivity in the context of very preterm births [5]. Also, using this protocol, a significant decrease in maternal stress and a lower risk of postpartum depression were found [6].

When combined with kangaroo care, maternal singing has a positive and significant impact on the autonomic stability of preterm infants [7]. Parental “Early Vocal Contact” [8] using singing and speech has also been highlighted as a promising approach in the NICU to promote preterm infants’ autonomic balance [8,9]. Live maternal singing enhances preterm infants’ vagal activity in the short term, thus improving neonatal autonomic stability [10].

The benefits of live music therapy using humming in a contingent way—synchronizing musical features with infants’ signals—were observed in early preterm infants’ self-regulation in the NICU [11,12,13,14,15,16,17].

Music therapy and sound healing based on the vibroacoustic effects of sounds are increasingly utilizing vocal toning including humming in clinical practice to promote deep relaxation and a change in the state of consciousness [18]. Vocal toning is a type of vocalization that uses the natural voice to express sounds including humming for the full exhalation of the breath and open vowel sounds [18], as well as “OM” chanting [19]. A study with adults observed that vocal toning can improve attention, awareness, and consciousness [18]. Also, “meditative”, “calm”, and “relaxed” states, which are associated with the parasympathetic nervous system and a decrease in HR, were the best descriptors of vocal toning. On the contrary, singing evoked emotions that were stronger than the emotions evoked by vocal toning; among those, the three most common descriptors were “nostalgia”, “tenderness”, and “joyful activation” [18].

After preterm birth, the maternal emotional state can make it difficult to communicate verbally with the infant, and, therefore, singing (with words) can evoke very intense emotions, while humming can facilitate the connection with the preterm infant in a safer way.

According NIDCAP approach [2], preterm infants’ vocal responsiveness can be considered an indicator of behavioral self-regulation if a balance is present in the relationship among the autonomic, the motor, and the states of consciousness subsystems. Otherwise, it may be interpreted as a behavior that may require too much effort and energy expenditure for preterm infants.

Vocal toning including humming improves cardiovascular function, respiratory flow, and diaphragmatic breathing [19,20,21]. These improvements have been identified as good psychophysiological indicators of decreased anxiety and increased relaxation [22]. The effectiveness of humming for improving preterm infants’ physiological parameters is associated with a decrease in HR to adequate values and an increase in O_2_ saturation [11,12,13].

Although no significant results were found during live music therapy for preterm infants’ physiological stabilization, a significant decrease in HR and deeper sleep were observed 30 min after the end of music therapy [23]. Also, live lullabies improved the sleep states of preterm infants more than recorded lullabies [24]. However, both conditions were associated with a decrease in the infants’ heart rate (HR), and no changes in the oxygen saturation level were observed [24]. This way, there were no significant differences in preterm infants’ physiological parameters in response to recorded lullabies compared to mothers’ live lullabies [25]. Lullabies, together with multimodal stimulation, induce benefits in weight gain/day and reduce the number of days of preterm infants’ hospitalization [26].

Potentially adverse effects of recorded maternal voice through infant directed speech (ID speech) and infant directed singing (ID singing) were found, expressed as decreased O_2_ saturation [27]. However, another study did not observe significant differences in HR or O_2_ saturation when comparing recorded maternal voice to the control condition [28]. A decrease in HR was found when the recorded maternal voice (speaking or reading) was delivered [29,30], while an increase in HR was found when a live maternal voice (speaking or singing) was directed to preterm infants [31].

Inconsistent results were observed about the effects of maternal voice for physiological outcomes such as HR, heart rate variability (HRV), and O_2_ saturation [7,11,26,27,30,32,33]. These inconsistencies may be due to methodological issues, namely the different conditions in which the maternal voice was delivered (recorded, live, speaking, reading, singing, humming, etc.). Most studies used a recorded maternal voice during a speech condition (speaking or reading), and few studies used a live maternal voice, either spoken or sung [8]. Few studies compared singing with speech, and very few studies used humming compared with singing. Very few studies in the field of music therapy in NICUs were found regarding the musical characteristics of singing or humming [12].

Maternal ID singing is a specific way for mothers to connect with infants that has a positive affect [34,35]. Despite dyads’ individual variations, maternal ID singing has universal characteristics that discriminate it from singing directed to adults. Studies of ID singing were conducted with term dyads, especially with infants older than three months [36]. Few studies regarding ID singing to preterm infants are reported in the literature [7,10,11,12,13,16].

Maternal ID singing is characterized by a slower tempo with a regular beat, a higher and consistent pitch level, and a more expressive vocal tone compared with adult-directed singing [36,37,38]. Many of these acoustic features are useful to capture the infants’ attention [34,35] and improve their self-regulation [39]. Remarkably, parents or mothers naturally change the way they sing based on infants’ needs and their behavioral states [40].

The lengthening of the final note in a musical phrase is a key feature of parental ID singing. This is a crucial temporal marker that helps infants to perceive units such as vocal phrases and phrase groupings [41,42,43]. It is likely that the lengthening of the final note in humming phrases is longer than in singing phrases, giving infants cues that help them to anticipate the end of humming phrases. In this vein, in a previous study carried out with the present sample, preterm infants’ overlapping vocalizations occurred predominantly during the lengthening of the final note in humming phrases [44]. This suggests that preterm infants can predict the end of humming phrases. Maternal breathing that follows the lengthening of the final note may be considered a physiological marker of the offset pauses between humming vocalizations. We think that this marker may induce the infants’ vocal response at the end of the final note.

Regarding the melodic contours of ID singing, Falk [45] observed six types of melodic contours: linear, rising, falling, bell-shaped, U-shaped, and sinusoidal contours. A microanalytical study with the present sample found a predominance of sinusoidal contours (29.92%), followed by bell-shaped (23.11%), rising (20.72%), falling (15.55%), U-shaped (6.70%), and linear contours (3.97%), in maternal humming directed to preterm infants [46]. In addition, the predominance of infants’ overlapping vocalizations during sinusoidal and bell-shaped contours was observed [46]. These melodic contours are predominantly observed in world lullabies characterized by smoothly oscillating sinusoidal contours, as well as descending, bell-shaped, and flat contours with a lengthening of the final note, where the pitch range tends to be smaller [47]. Infant-directed lullabies aim to calm by decreasing the arousal level, ensuring an intimate and warm environment, and inducing a sleep state [40].

It is important to understand if maternal humming during kangaroo care is able to improve physiological synchrony in preterm dyads. In short, does the regularity of humming in a lullaby helps to stabilize mothers’ cardiorespiratory function and maintain it in a relaxation state during kangaroo care? More than that, does maternal relaxation through humming improve the infants’ self-regulation?

### Purpose of the Present Study

As a vibroacoustic experience that improves vagal activity [19], maternal humming during skin-to-skin contact may be even more effective than speech at enhancing the parasympathetic responses associated with the maternal relaxation state. Compared with speech, humming is associated with improvements in maternal respiratory flow and cardiovascular function, with a lower vocal intensity and a smaller tonal variation, which may improve preterm infants’ self-regulation.

Our study focused on humming’s effects upon the physiological parameters of preterm dyads during kangaroo care. Among several aspects, we think that humming musical features such as melodic contours and the lengthening of the final note contribute to its effectiveness. There is a gap in research about the musical characteristics of humming produced during music therapy sessions. Therefore, it is essential to know how the musical features of humming can help to improve preterm infants’ physiological stability. This will be useful for preterm mothers’ guidance while improving humming aimed at their infants during kangaroo care.

Infants’ physiological stability is ensured when an adequate level of oxygen (O_2_) saturation is achieved with less effort in heart rate (HR) and breathing [1]. Thus, a new physiological measure such as the proportion of O_2_ saturation relative to HR (Prop. O_2_ saturation/HR) should be considered to evaluate the effects of stimulation on preterm infants’ cardiorespiratory stability.

The aims of the present study are to evaluate the following aspects during kangaroo care: (1) the effect of maternal humming vs. speech on preterm infants’ physiological parameters (infants’ HRs and infants’ Prop. O_2_ saturation/HR ratios); (2) the effect of maternal humming vs. speech on mothers’ physiological parameters (HR and O_2_ Saturation); (3) the impacts of humming melodic contours (linear, rising, falling, bell-shaped, U-shaped, and sinusoidal contours) and the lengthening of the final note on preterm infants’ physiological parameters.

## 2. Method

### 2.1. Design

The present study is a single-group repeated measures design included in a broader study of preterm infants’ vocal responsiveness in humming vs. speech conditions during kangaroo care [44,46,48], and it was approved by the Ethics Committee of the Hospital [48]. Due to cultural and occupational issues, most of the fathers were not present in the NICU during the observations. So, only mothers were recruited for this study. All mothers gave written informed consent and were interviewed via a sociodemographic and clinical questionnaire.

According to the study protocol, each preterm dyad was observed only once in the NICU during kangaroo care in a sequence composed of silent and vocal conditions (humming and speech). The skin-to-skin contact followed the KSDFP instructions [4], which aimed to improve visual interaction between mother and infant. For this, a large scarf was provided with an additional strap for the support of the infant’s neck. Mothers were invited to address their infants by speaking (as usually) and humming (humming an improvised melody in a spontaneous way with the mouth closed) while in kangaroo care. Also, no guidance was given to mothers regarding the use of musical repertoire, and there was no support or orientation from a music therapist. The protocol alternated between each vocal condition and silent condition during 5 periods that were each 3 min long: (1) silent baseline, (2) maternal voice (speech or humming), (3) silence, (4) maternal voice (humming or speech), and (5) final silence. In the baseline condition, mothers were silent, and other modalities of maternal contact were not discouraged (gaze, touch, rocking, etc.). The order effect was controlled; after the silent baseline, mothers assigned with odd numbers started with the humming condition, and mothers assigned with even numbers started with the speaking condition.

All observations occurred at the same time of day 10 min after the infant had been placed in kangaroo care and before being fed. If the infant presented a state of restlessness or crying, we waited for the infant to enter a state of quiet alertness or drowsiness [49] (pp. 49–51) in order to ensure that they would be available for the interaction. All observations took place in the neonatal intermediate care unit, a room where 6 incubators were isolated from external noises by a closed sliding door. Only the mother with the infant, the researcher who recorded the observations on video, and the nursing team were present. Before (three hours of sleep) and during the observation protocol, painful procedures were not performed. If critical episodes arose, nurses would intervene, and the protocol would be interrupted. The setting of the preterm infant according to the KSDFP was exclusively performed by the nurses.

All observations of the dyads were audio- and video-recorded. For this, two cameras (Panasonic 4K HC-VX870, Junichi Suzuki, Panasonic Europe B.V. WTC H-16, Zuidplein 136, 1077XV Amsterdam, The Netherlands) were used. The first camera was directed to the mother’s and infant’s faces in order to capture both individuals’ faces simultaneously in real time. To measure the vocalizations of the dyad, an external microphone was connected to the first camera. Video (MP4) and audio (WAV) recordings were made for the mother’s and infant’s vocalizations during speech and humming. For the present study, to analyze the dyads’ physiological parameters, a second camera was directed to the NICU’s monitors to observe the values of each infant’s physiological parameters (O_2_ saturation level and HR) and, at the same time, to the mother’s oximeter (O_2_ saturation level and HR) during several conditions. Before initiating the video recording, the oximeter was attached to the index finger of each mother.

### 2.2. Measures

A sociodemographic and clinical questionnaire was used to collect basic information about participating dyads and their obstetric and pediatric backgrounds. ELAN software (EUDICO Linguistic Annotator version 4.9.4) was used to code the data (maternal humming, maternal speech, and physiological parameters of both partners) for microanalytical analyses.

#### 2.2.1. Recording, Minute-by-Minute, Mothers’ and Infants’ HRs and O_2_ Saturation

To observe the effects of humming compared to maternal speech on the dyads’ physiological parameters, values of HR and O_2_ saturation (displayed on the NICU’s monitors and on the mother’s oximeter) were recorded minute by minute throughout the observation (at the first, second, and third minutes during the humming and speech periods) using ELAN software. We also estimated the Prop. O_2_ saturation/HR ratio of the mother, as well as of the infant, in each condition (silent baseline, humming, and speech) to assess the cardiac effort needed to reach an optimal level of O_2_ saturation. The means of the three values were estimated for HR, O_2_ saturation, and Prop. O_2_ saturation/HR ratio in each condition.

#### 2.2.2. Maternal Vocalizations during the Protocol

In a broader study [48], all maternal vocalizations during humming and during speech were coded (the frequency of vocalizations per minute and duration of each vocalization in milliseconds). According to the criteria of Gratier and team [50], each maternal vocalization was identified as the production, by the mother, of an audible vocal sound without interruptions exceeding 300 ms. The first author was trained at the Baby Lab of the Université Nanterre La Defense for the management of ELAN software. In order to achieve reliability, a second researcher, trained by the first author, participated in data coding.

#### 2.2.3. Humming Melodic Contours and the Lengthening of the Final Note

With the same sample, a previous study coded the melodic contours, as well as the lengthening, of the final note of maternal vocalizations (N = 1761) during humming [46]. The coding was based on the classification of the six tonal contours developed by Falk [45]: (1) the linear contour, when the humming vocalization was flat (the vocalization presents only one musical note); (2) the rising contour, if there was a tonal increase; (3) the falling contour, characterized by a tonal decrease; (4) the bell-shaped contour, characterized by an increase followed by a decrease; (5) the U-shaped contour, characterized by a decrease followed by an increase; and (6) the sinusoidal contour, a bell-shaped contour, followed by a rising contour or a U-shaped contour, in turn followed by a falling contour.

ELAN software was used to code the melodic contours (frequency and duration of each humming vocalization), as well as to code the lengthening of the final note of melodic contours (duration of the final note in each humming vocalization), in the audio and video recordings. For this, there were two researchers (the first author and a student of musicology), both with extensive academic musical training (8th degree of musical training). They listened (simultaneously visualizing the audio-spectrogram displayed using ELAN software) to the humming of each mother to identify the type, according to the Falk protocol, [45] and duration of the melodic contour for each maternal vocalization. Also, the duration of the lengthening of the final note in each humming vocalization was coded in ELAN software by the same researchers.

### 2.3. Sample Selection Process

This study was carried out with a sample of preterm dyads in the NICU of the Hospital [48]. Due to difficulties involved in recruiting participants in the clinical context of prematurity, we intended to select 50 dyads. Several unexpected factors meant that only 36 dyads could be used for this study.

While recruiting dyads, the following exclusion criteria were applied to mothers: (a) a maternal age under 19 years old, (b) difficulty understanding and speaking the Portuguese language, (c) the presence of a maternal auditory deficit, (d) the absence of medical supervision during gestation, (e) a history of psychiatric pathology or existence of serious negative emotional states, and (f) addictions. Also, dyads were excluded if infants (a) had a post-menstrual age of less than 32 weeks or more than 37 weeks, (b) presented unstable vital parameters, (c) needed the support of CIPAP (Continuous Positive Airway Pressure), (d) had intraventricular hemorrhages, (e) had congenital or neurological anomalies of the auditory cortex, (f) had a nasogastric tube, and (g) needed their breathing to be supported. Dyads were also excluded if kangaroo care had not been practiced at least once previously.

### 2.4. Participants

Table 1 displays the sociodemographic and clinical data of dyads regarding maternal age, education, and nationality, as well as infants’ gender, gestational age at birth, chronological age at observation, weight at birth, and weight at observation. Most of the mothers are Portuguese nationals or African and Brazilian mothers who have lived in Portugal for at least 7 years. None of the mothers had received formal musical training. Most infants are males.

### 2.5. Reliability

For the broader study, two independent researchers coded 30% of all mothers’ vocalizations using Elan software to quantify the number of vocalizations—vocal segments before and after pauses—in humming and speech [48]. The independent researchers were blind to the vocal conditions. Intraclass correlation coefficients (ICC) were used to assess differences between the researchers. Good agreement was found for the number of maternal humming vocalizations (ICC = 0.999, *p* = 0.000), as well as for the number of maternal speech vocalizations (ICC = 0.991, *p* = 0.000).

Regarding the melodic contours of humming, two researchers with a musical background (the first author and a student of musical sciences with musical training and experience in music analysis) also carried out a reliability analysis of the melodic contours of humming [46]. They studied seven dyads (19.44% of the participating dyads) and achieved a high degree of agreement for all melodic contours: (1) linear (ICC = 0.944, *p* = 0.002), (2) rising (ICC = 0.999, *p* < 0.001), (3) falling (ICC = 0.996, *p* < 0.001), (4) bell-shaped (ICC = 0.995, *p* < 0.001), (5) U-shaped (ICC = 0.933, *p* = 0.002), and (6) sinusoidal (ICC = 0.992, *p* < 0.001) contours.

## 3. Results

A descriptive statistical analysis was performed using SPSS 27 software for preterm dyads’ physiological variables (HR, O_2_ saturation, and Prop. O_2_ saturation/HR ratio) during the silent baseline, humming, and speech conditions. Also, comparative statistical analyses (Student’s *t*-test) for paired samples were performed to identify significant differences between the physiological parameters (HR, O_2_ saturation, and the Prop. O_2_ saturation/HR ratio) for each partner during the silent baseline, humming, and speech conditions.

Regarding the effects of the durations of the melodic contours (linear, rising, falling, bell-shaped, U-shaped, and sinusoidal contours) and the lengthening of the final note on infants’ physiological parameters during humming, several multiple hierarchical linear regressions were performed.

Table 2 presents the descriptive statistics of mothers’ HRs, O_2_ saturation, and Prop. O_2_ saturation/HR ratios relative to three-minute periods in each condition. According to our results, a higher level in mothers’ HRs is observable for both the humming and speech conditions; the highest value is observed during the speech condition. Mothers’ O_2_ saturation values change slightly across the protocol conditions.

**Figure 1:**
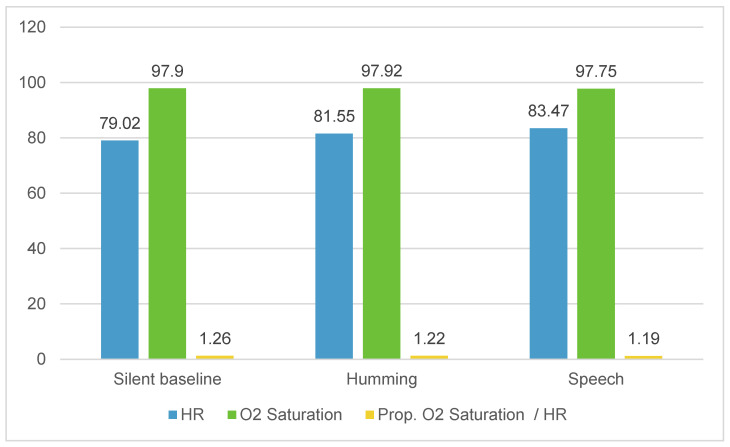
Maternal levels of physiological variables (HR, O_2_ saturation, Prop. O_2_ saturation/HR ratio) during silent baseline, humming and speech conditions.

In Figure 1, it is possible to observe the maternal levels of the physiological parameters according to the vocal conditions.

Table 3 presents the descriptive statistics of infants’ HRs, O_2_ saturation, and Prop. O_2_ saturation/HR ratios for the several three-minute periods in each condition. According to our results, the HR mean is lower during the humming condition, reaching its highest point during the speech condition. Infants’ O_2_ saturation seems to remain stable.

**Figure 2:**
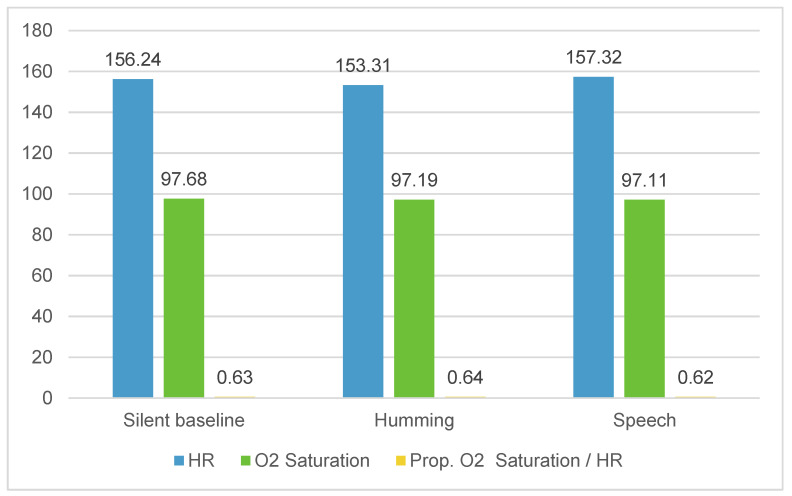
Infants’ levels of physiological variables (HR, O_2_ saturation, Prop. O_2_ saturation/HR ratio) during silent baseline, humming and speech conditions.

In Figure 2, it is possible to observe the infants’ levels for the physiological parameters according to the vocal conditions.

### 3.1. Comparison of Outcomes across Conditions

Table 4 displays the significant differences in the mothers’ physiological variables. According to the results, we found a significantly higher mean HR during humming (t = −4.889, df = 33, *p* < 0.001, Cohen’s d = −0.839), as well as during speech (t = −5.397, df = 33, *p* < 0.001, Cohen’s d = −0.926), compared to the silent baseline. Both significant differences have a very large effect size according to Cohen’s d statistics. However, only a slight difference was found between maternal HR in humming and speech (t = −2.037, df = 32, *p* = 0.050, Cohen’s d = −0.355), with a higher mean HR in speech than humming. This difference presents a medium effect size for Cohen’s d statistics. For Prop. O_2_ saturation/HR, significant differences with Cohen’s d statistics ranging from medium to very large effect sizes were found between (1) the silent baseline and humming (t = 5.060, df = 33, *p* < 0.001, Cohen’s = 0.868), with a higher Prop. O_2_ saturation/HR ratio in the silent baseline than humming; (2) the silent baseline and speech (t = 5.511, df = 33, *p* < 0.001, Cohen’s = 0.945), with a higher Prop. O_2_ saturation/HR ratio in the silent baseline than in speech; and (3) humming and speech (t = 2.289, df = 32, *p* = 0.029, Cohen’s = 0.398), with a higher Prop. O_2_ saturation/HR ratio in humming than speech. These significant differences must be taken into account once their effects present values varying from medium to very large.

Optimal values in mothers’ O_2_ saturation (M = 97, min. = 94, max. = 99) were found for all conditions, and no significant differences were observed.

Table 5 displays the significant differences in the infants’ physiological variables. According to the results, the mean HR was lower during humming (M = 153.315) compared to speech (M = 157.324), and this difference is statistically significant, presenting a medium effect size according to Cohen’s d statistics (t = −2.293, df = 35, *p* = 0.028, Cohen’s = −0.382). No significant differences were found for infants’ means of O_2_ saturation between the speech and humming conditions. Also, O_2_ saturation presents optimal values (M = 97; min. = 88; max. = 100) in both conditions. However, the Prop. O_2_ saturation/HR ratio is higher during humming than speech, and this difference is significant (t = 2.309, df = 34, *p* = 0.027). Also, this difference needs to be considered since its effect size is medium in Cohen’s d terms (Cohen’s d = 0.390).

### 3.2. Effects of Melodic Contours and Lengthening of the Final Note on Preterm Infants’ Hysiological Parameters

Table 6 displays the single significant result obtained for preterm infants’ HRs during humming as a dependent variable and mean durations of sinusoidal contours and mean lengthening of the final notes as independent variables. Using non-sinusoidal contours (linear, falling, rising, bell-shaped, and U-shaped contours) together with the lengthening of the final note as independent variables did not yield any significant results. As sociodemographic and clinical variables of preterm infants may influence their HRs, in Model 1, infants’ gestational ages at birth, infants’ chronological ages at observation, and infants’ gender parameters were included as predictor variables. As an infant’s physiological system may be deeply connected to their mother’s physiological system during kangaroo care, maternal physiological parameters (mothers’ mean HRs in humming and mothers’ mean O_2_ Saturation in humming) were included as predictor variables in Model 2. Finally, the mean durations of each melodic contour during humming (linear, rising, falling, bell-shaped, U-shaped, and sinusoidal contours), as well as the mean lengthening of the final notes in humming, both as independent variables, were included in Model 3. According to the entry method, Model 2 included all independent variables of Model 1, and Model 3 included all independent variables present in Model 2. According to Table 6, independent variables included in Model 1 and Model 2 do not seem to contribute to the explanation of the variance of the dependent variable. However, relative to Model 3, a significant contribution is found. According to our results, only the mean duration of humming sinusoidal contours, together with the lengthening of the final notes, impacts preterm infants’ HRs during humming (*p* = 0.025).

Table 7 shows results for preterm infants’ Prop. O_2_ saturation/HR ratios during humming as a dependent variable and mean durations of each melodic contour of humming as a principal independent variable. As sociodemographic and clinical variables of preterm infants may influence preterm infants’ Prop. O_2_ saturation/HR ratios, infants’ gestational ages at birth, infants’ chronological ages at observation, and infants’ gender parameters were included as predictor variables in Model 1. For the same reason presented in the previous analyses, maternal physiological parameters (mothers’ mean HRs in humming and mothers’ mean O_2_ saturation in humming) were included as predictor variables in Model 2. Finally, the mean durations of each melodic contour during humming (linear, rising, falling, bell-shaped, U-shaped, and sinusoidal contours), as well as the mean lengthening of the final notes in humming, both as independent variables, were included in Model 3. In the same manner as in the previous regression analysis, the entry method was used, and, therefore, the independent variables of each model were included in the next model. According to our results, the variables of Model 1 and Model 2 do not contribute to explaining the variance of the dependent variable. In contrast, the predictors variables of Model 3 offer a significant contribution for the explanation of the dependent variable (*p* = 0.037). The mean duration of sinusoidal contours, together with the mean lengthening of the final notes in humming, can be considered a good predictor of the infants’ Prop. O_2_ saturation/HR ratios in humming.

## 4. Discussion

To date, according to our knowledge, this is probably a pioneering study, as it aimed at understanding the effects of melodic contours and the lengthening of the final note in maternal ID humming on preterm infants’ autonomic systems during kangaroo care. Regarding physiological measures used to assess preterm infants’ ANS (HR mean, HR range, HRV and O_2_ saturation), there are defined as highlighted in the past state of the art studies [7,11,17,26,28,30,32,33]. Our study added the infants’ Prop. O_2_ saturation/HR ratios as a new and promising physiological measure for this research field. This was based on the idea that autonomic stability can be improved if it is achieved while preserving infants’ cardiac resources; this is relevant to ensure preterm infants’ physiological stability [1].

Regarding the first aim of this study, our results show the lower level (group mean) of preterm infants’ HRs during maternal humming than maternal speech. Also, a higher level (group mean) of infants’ Prop. O_2_ saturation/HR ratios was found during humming compared with speech. Therefore, the level of the Prop. O_2_ saturation/HR ratio shows that the optimal value of O_2_ saturation can be achieved with a lesser cardiac effort. This suggests that humming is a favorable condition for decreasing preterm infants’ HRs while, at the same time, O_2_ saturation reaches an optimal level.

Regarding the second aim, a higher maternal Prop. O_2_ saturation/HR ratio in humming than speech was found. This suggests that mothers’ humming during kangaroo care may improve maternal cardiorespiratory function, as highlighted in studies about the physiological benefits of vocal toning in adults [18,19,20,21].

Concerning the third aim of this study, it was observed that the mean duration of humming sinusoidal contours, together with the lengthening of the final notes, helps to decrease preterm infants’ HR. Furthermore, the stability of O_2_ saturation is achieved with a lesser cardiac effort. Once it is known that O_2_ saturation is crucial for human infants’ viability, and that HR is indispensable to achieve its’ regulation [1], it is important to relate both variables. As preterm infants are particularly vulnerable to cardiac efforts, O_2_ saturation should not be considered in the absence of HR.

Despite mothers not being asked to hum lullabies or hum in a lullaby style, our results show that maternal humming presents structural and melodic features of that style. Sinusoidal contours and the lengthening of the final notes are usually observed in a lullaby style [47]. As highlighted by music therapy in the NICU [11,12,13,14,15], our results suggest that maternal humming in a lullaby style can improve preterm infants’ physiological stability.

Concerning infants’ vocal responsiveness, in a previous study conducted with the same sample. it was observed that more than half of the infants’ overlapping vocalizations took place during the final note [51]. Probably, the lengthening of the final note can be perceived by the infants as a temporal marker, helping them to respond during the end of the humming phrase.

As mentioned in the literature, humming has a strong effect on the vagal system [19], inducing a relaxation state, and wellbeing, releasing endorphins, enhancing sleep, lowering the heart rate and blood pressure, decreasing cortisol, and increasing oxytocin [52]. Therefore, humming in a lullaby style attuned to preterm infants’ behavior is commonly used as a music therapy in the NICU [11,12,13,14,15].

Our study highlights the important role of the musical characteristics of humming that has been overlooked in studies of music therapy and other neonatal stimulation approaches directed at preterm infants in the NICU. When mothers hum to their preterm infants’ during kangaroo care, guidance about humming features should be offered to optimize infants’ self-regulation. Maternal humming is likely an easy way to improve the mother–infant relationship during kangaroo care. If maternal humming in kangaroo care is offered in a lullaby style and by keeping the voice calm, slow, simple, predictable, and repetitive, it can optimize the cardiorespiratory functioning of both the mother and preterm infant [12].

When the infant is agitated and presenting tachycardia, maternal humming should be repetitive, consisting of continuous notes with short intervals, predominantly with sinusoidal or descending melodic contours and the repetition of the final notes or their lengthening. On the other hand, it is important that these guidelines do not induce an excessively relaxed state that could induce severe bradycardia. This reinforces the use of contingent singing including humming in music therapy applied to NICU contexts [12,13,14,15].

The joint effect of O_2_ saturation with HR assessed based on the Proportion of O_2_ saturation/HR is a promising physiological variable that should be considered in future studies to clarify the benefits of maternal humming in preterm infants’ cardiorespiratory stabilization.

In an opposite sense, other studies using maternal singing presented different results relative to the impact on infants’ physiological parameters. One of these studies [26] only observed a decrease in HR 30 min after the end of the intervention. In another study [53], maternal singing during feeding did not have any impact on infants’ physiological results. It should be stressed that both these studies used maternal singing and not humming. In future studies, it would be important to compare the effects of humming versus singing on preterm infants’ physiological parameters.

### Limitations

Our study has several limitations. One of these is that we did not analyze the impact of the NICU’s noise, which could have affected the physiological parameters of the dyads. Although infants’ behavioral states and clinical status were controlled as baseline conditions, these aspects were not monitored during the protocol sequence. It is possible that these factors were affected by the protocol conditions and, in this way, changed the infants’ physiological parameters. In addition, mothers varied their tactile behavior during the interaction, touching and caressing the infant while they spoke or sang and during silent pauses. The possible effects of maternal touch and rocking directed at the infant during humming were not analyzed. Finally, we did not analyze the maternal emotional state during the observation, which is another factor that could have affected mothers’ vocal expressiveness, as well as their physiological parameters.

Following studies using multimodal stimulation in music therapy programs with preterm infants [26,54], it is important to deepen the joint effect of humming with kangaroo care on infants’ self-regulation. Among multimodal stimulations, there are rocking, and touch coordinated with rhythmic features of maternal humming/singing during kangaroo care, i.e., rocking associated with a vestibular experience that can have an impact on the preterm infants’ ANSs.

## 5. Conclusions

Our study contributes to the current literature on maternal humming effects in NICUs, underlining the important contribution of the musical features of humming such as sinusoidal contours and the lengthening of the final note to preterm infants’ physiological parameters.

Maternal humming can improve maternal cardiorespiratory function and, therefore, should be recommended in NICUs as an easy personal resource to foster maternal well-being and enable preterm infants’ self-regulation. The use of maternal contingent humming in a lullaby style during kangaroo care in NICUs should be encouraged. More studies are needed to deepen our preliminary results.

## Figures and Tables

**Table 1:** Descriptive statistics of sociodemographic and clinical data of preterm dyads (N = 36).

Variables	n	%	M	SD	Min.–Max.
maternal age			34.20	5.63	21–48
maternal education			15.33	3.69	6–24
Portuguese nationality	26	72.22			
African and Brazilian nationality	10	27.78			
male infants	20	55.55			
female infants	16	44.44			
infants’ gestational age at birth *			30.40	2.44	25.42–34.42
infants’ chronological age at observation **			26.5	19.99	4–81
infants’ weight at birth (g)			1265.47	308.20	590–2017
infants’ weight at observation (g)			1538.05	237.72	1060–2185

* Number of weeks; ** number of days.

**Table 2:** Descriptive statistics of mothers’ physiological variables (HR *, O_2_ saturation **, Prop. O_2_ saturation/HR ratio ***) during silent baseline, humming and speech conditions.

Physiological Parameters	Silent Baseline		Humming		Speech	
	M (SD)	Min.–Max.	M (SD)	Min.–Max.	M (SD)	Min.–Max.
HR *	79.02 (10.17)	47.67–97.67	81.55 (10.75)	49.00–101.00	83.47 (10.39)	50.67–101.00
O_2_ saturation **	97.90 (1.14)	94.00–99.00	97.92 (1.10)	94.33–99.00	97.75 (1.14)	94.33–99.00
P. O_2_ S/HR ***	1.26 (0.19)	0.99–2.06	1.22 (0.19)	0.95–2.00	1.19 (0.18)	0.95–1.93

* Heart rate; ** oxygen saturation; *** proportion of oxygen saturation relative to heart rate.

**Table 3:** Descriptive statistics of infants’ physiological variables (HR *, O_2_ saturation **, Prop. O_2_ saturation/HR ratio ***) during silent baseline, humming, and speech conditions.

Physiological Parameters	Silent Baseline		Humming		Speech	
	M (SD)	Min.–Max.	M (SD)	Min.–Max.	M (SD)	Min.–Max.
HR *	156.24 (11.37)	137.00–181.00	153.31 (13.54)	119.00–185.67	157.32 (12.08)	130.00–188.00
O_2_ saturation **	97.68 (3.33)	86.36–100.00	97.19 (3.15)	88.00–100.00	97.11 (3.02)	88.00–100.00
P. O_2_ S/HR ***	0.63 (0.05)	0.52–0.73	0.64 (0.06)	0.50–0.74	0.62 (0.06)	0.51–0.77

* Heart rate; ** oxygen saturation; *** proportion of oxygen saturation relative to heart rate.

**Table 4:** Comparative statistics between mothers’ physiological variables (HR *, O_2_ saturation **, Prop. O_2_ saturation/HR ratio ***) during silent baseline (BL), humming (Hum), and speech (Sp) conditions.

Physiological Parameters	Comparisons	t	df	*p*	Cohen’s d
HR *	BL vs. Hum	−4.89	33	<0.001	−0.839
	BL vs. Sp	−5.39	33	<0.001	−0.926
	Hum vs. Sp	−2.04	32	0.050	−0.355
O_2_ saturation **	BL vs. Hum	−0.11	33	0.917	−0.018
	BL vs. Sp	1.59	33	0.121	0.273
	Hum vs. Sp	1.75	32	0.089	−0.355
Prop. O_2_ saturation/HR ratio ***	BL vs. Hum	5.06	33	<0.001	0.868
	BL vs. Sp	5.51	33	<0.001	0.945
	Hum vs. Sp	2.29	32	0.029	0.398

* Heart rate; ** oxygen saturation; *** proportion of oxygen saturation as a function of heart rate. Very small (0.0–<0.1); small (0.1–<0.30); medium (0.30–<0.50); large (0.50–<0.80); very large (0.80–<1.20); huge (1.20–2.0).

**Table 5:** Comparative statistical analyses between infants’ physiological variables (HR *, O_2_ saturation **, Prop. O_2_ saturation/HR ratio ***) during the silent baseline (BL), humming (Hum), and speech (Sp) conditions.

Physiological Parameters	Comparisons	t	df	*p*	Cohen’s d
HR *	BL vs. Hum	1.62	35	0.114	0.270
	BL vs. Sp	−0.95	35	0.349	−0.158
	Hum vs. Sp	−2.29	35	0.028	−0.382
O_2_ saturation **	BL vs. Hum	1.06	35	0.297	0.177
	BL vs. Sp	1.32	34	0.195	0.224
	Hum vs. Sp	0.00	34	1.000	0.000
Prop. O_2_ saturation/HR ***	BL vs. Hum	−1.26	35	0.218	−0.209
	BL vs. Sp	1.37	34	0.179	0.232
	Hum vs. Sp	2.31	34	0.027	0.390

* Heart rate; ** oxygen saturation; *** proportion of oxygen saturation relative to heart rate. Very small (0.0–<0.1); small (0.1–<0.30); medium (0.30–<0.50); large (0.50–<0.80); very large (0.80–<1.20); huge (1.20–2.0).

**Table 6:** Multiple linear hierarchical regression for DV—infants’ HRs during humming—and IVs—the average duration of sinusoidal contours and lengthening of the final notes.

Model	R	R^2^	Adjusted R^2^	St. Error of Estimate	R^2^ Change	F Change	df1	df2	Sig. of F Change
1	0.331	0.109	0.020	13.186	0.109	1.228	3	30	0.317
2	0.336	0.113	−0.046	13.624	0.003	0.051	2	28	0.951
3	0.575	0.331	0.151	12.275	0.218	4.246	2	26	0.025

HR: heart rate; Model 1: infants’ gestational ages at birth, infants’ chronological ages, and infants’ genders; Model 2: mothers’ HRs in humming and mothers’ O_2_ saturation in humming; Model 3: the average lengthening of the final notes and average duration of humming sinusoidal contours; DV: dependent variable; IVs: independent variables.

**Table 7:** Multiple linear hierarchical regression for the DV—infants’ Prop. O_2_ saturation/HR ratios during humming—and IVs—the average duration of sinusoidal contours and lengthening of the final notes.

Model	R	R^2^	Adjusted R^2^	St. Error of the Estimate	R^2^ Change	F Change	df1	df2	Sig. of F Change
1	0.369	0.136	0.050	0.051	0.136	1.574	3	30	0.216
2	0.395	0.156	0.005	0.052	0.020	0.327	2	28	0.724
3	0.587	0.345	0.168	0.048	0.189	3.749	2	26	0.037

Proportion of O_2_ saturation relative to HR; Model 1: infants’ gestational ages at birth, infants’ chronological ages, and infants’ gender; Model 2: mothers’ HRs in humming and mother’s O_2_ saturation in humming; Model 3: average lengthening of the final note and average duration of the humming sinusoidal contours; DV: dependent variable; IVs: independent variables.

## Data Availability

The data presented in this study are available on request from the corresponding author. The data are not publicly available due to privacy.
